# Factors associated with cervical cancer screening participation among immigrants of Russian, Somali and Kurdish origin: a population-based study in Finland

**DOI:** 10.1186/s12905-017-0375-1

**Published:** 2017-03-11

**Authors:** Esther E. Idehen, Tellervo Korhonen, Anu Castaneda, Teppo Juntunen, Mari Kangasniemi, Anna-Maija Pietilä, Päivikki Koponen

**Affiliations:** 1 Institute of Public Health and Clinical Nutrition, University of Eastern Finland, Yliopistoranta 1, P. O. Box 1627, 7021 Kuopio, Finland; 2 Department of Public Health, University of Helsinki, Helsinki, Finland; 3 Department of Welfare, Equality and Inclusion Unit, National Institute for Health and Welfare (THL), Helsinki, Finland; 4 Department of Nursing Science, University of Eastern Finland, Kuopio, Finland; 5 Department of Public Health Solutions, National Institute for Health and Welfare (THL), Helsinki, Finland

**Keywords:** Cervical cancer, Early detection, Finland, Immigrants, Pap test, Screening participation

## Abstract

**Background:**

Previous studies revealed low participation in cervical cancer screening among immigrants compared with non-immigrants. Only a few studies about factors associated with immigrants’ lower participation rates have been conducted in European countries that have universal access for all eligible women. Our study aimed to explore factors associated with cervical screening participation among women of Russian, Somali, and Kurdish origin in Finland.

**Methods:**

We used data from the Migrant Health and Well-being Survey, 2010-2012. Structured face-to-face interviews of groups of immigrants aged 25-60 yielded 620 responses concerning screening participation in the previous five years. Statistical analysis employed logistic regression.

**Results:**

The age-adjusted participation rates were as follows: among women of Russian origin 73.9% (95% CI 68.1-79.7), for Somalis 34.7% (95% CI 26.4-43.0), and for Kurds 61.3% (95% CI 55.0-67.7). Multiple logistic regressions showed that the most significant factor increasing the likelihood of screening participation among all groups was having had at least one gynecological check-up in the previous five years (Odds ratio [OR] = 6.54-26.2; *p* < 0.001). Other factors were higher education (OR = 2.63; *p* = 0.014), being employed (OR = 4.31; *p* = 0.007), and having given birth (OR = 9.34; *p* = 0.014), among Kurds; and literacy in Finnish/Swedish (OR = 3.63; *p* = 0.003) among Russians.

**Conclusions:**

Our results demonstrate that women who refrain from using reproductive health services, those who are unemployed and less educated, as well as those with poor language proficiency, might need more information on the importance of screening participation. Primary and occupational healthcare services may have a significant role in informing immigrant women about this importance.

## Background

Cervical cancer is one of the most common cancers among women worldwide and thus a significant public health problem [1]. Globally, about 528,000 new cases of, and 266,000 deaths from cervical cancer were reported in 2012; approximately 87% of those occurred in developing countries [2]. This disease can be detected early by regular Smear (Pap) tests, widely used to screen women for the disease through organized or opportunistic screening programs [3]. The World Health Organization recommends cervical cancer screening to all women as most of those at risk might be asymptomatic [4]. The effectiveness of the screening in most developed countries is sufficiently evident. Appropriate screening and health education programs have reduced both the incidence and mortality rates [5, 6]. Previous studies highlight that, despite the availability and effectiveness of this screening in the most developed countries, a high proportion of cervical cancer patients had irregular or no screening [7, 8].

Previous studies reported immigrants’ low cervical cancer screening participation compared with non-immigrant women in different countries [9–11], indicating that the screening participation varies within and across various ethnic groups. Several factors account for low screening participation among immigrants; examples are screening ineffectiveness, inaccessibility of healthcare services, unaffordable medical treatments, lack of awareness of screening, and risk of cervical cancer [1, 12–14]. Other barriers are poor language proficiency, lack of trust in healthcare services, experiences of discrimination [15, 16], perceived embarrassment or anxiety with Pap test procedures, or Female Genital Mutilation (FGM) experiences [17]. Factors such as higher education level, being married or in common law relationship, younger age of migration and longer stay in the host country [9, 11, 18, 19], being employed, having given birth, and higher number of family members, [20–23] increase the likelihood of screening participation among immigrants.

Finland has a focus on preventive care and public healthcare services covering the entire population of approximately five million. The goal is to ensure equal accessibility to healthcare for all inhabitants. Since the 1960s, a nation-wide ‘cost-free’ organized cervical cancer screening program has targeted all women in selected age groups (30 to 60) using a five-year interval; some municipalities target women aged 25 to 65 [3]. Women are identified from the national population register and receive personal invitation letters [24]. Among the general Finnish population, the screening participation rate is currently about 70% of all women invited to the systematic screening [3]. They may also have opportunistic (non-organized) tests taken on their own initiative or with a referral from their healthcare providers. Only a few studies exist about cervical cancer screening participation among immigrants in other European countries (Sweden, Norway, and the Netherlands) that have equally universal access for all eligible women [11, 20, 25, 26].

As the population of minority women increases, it is imperative to assess factors associated with screening participation, i.e. enabling factors and barriers for participation among different immigrant women living in Finland. The number of immigrants including females in Finland has increased in recent years [27]. Previous studies in Finland revealed lower participation rates for cervical cancer screening among immigrants compared with the general Finnish population [28]. To date, however, the reasons for this low participation have been unexplored. The aim of our study is to explore factors associated with cervical screening participation among women of Russian, Somali, and Kurdish origin in Finland. Such knowledge is necessary for developing efficient public health screening programs and appropriate allocation of resources. The goal is to enhance intervention strategies for increasing cervical cancer screening participation among immigrants.

## Methods

### Study population

Our data came from the Migrant Health and Well-being Survey, 2010-2012, of the Finnish National Institute for Health and Welfare (THL. The sample included 3000 immigrants, 1000 from each of the three countries of origin, i.e. those from Russia or the Soviet Union, Somalia, and Kurds from Iraq or Iran [29].The three major migrant groups were selected to represent different geographical areas. Russians were the largest, Somalis were the fourth largest, and Kurds were the sixth largest groups of the migrants in Finland listed by foreign language. The latter are Iraqi and Iranian refugees who have been among the largest groups of quota refugees accepted into Finland in recent years [30]. The Somali group were mainly refugees and Muslims. The sample was stratified by municipality and ethnic groups and randomly drawn from the Finnish National Population Registry. The inclusion criteria were native language, the above-mentioned countries of origin, age 18-64, having lived in Finland for at least one year, and living in one of the six cities with high proportions of immigrants: Helsinki, Espoo, Vantaa, Turku, Tampere, and Vaasa. For one part of the study protocol, the participation rates were as follows: Russians 70%, Somalis 51%, and Kurds, 61%. We obtained responses to the question about cervical cancer screening (Pap test) participation in the previous five years from 620 women (291 Russians, 132 Somalis, 197 Kurds) in the age group 25-60, the target age group for the Finnish national screening program.

### Data collection and variables

**Fig. 1:**
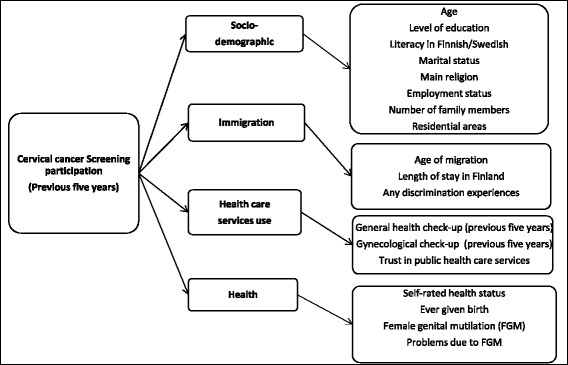
The dependent variable and potential explanatory variables in four main categories

Trained bilingual interviewers conducted structured face-to-face interviews, using the native languages of the participants. These interviews covered several topics on health, well-being and health service utilization. The dependent variable was self-reported participation in screening in the previous five years in Finland; answers were recorded as ‘yes’ or ‘no,’ without distinguishing whether such tests were opportunistic or taken as part of an organized screening program. Based on previous studies on potential factors associated with cervical cancer screening [9–12, 17, 21, 31], we divided the explanatory variables into four main categories. They were socio-demographic factors and variables related to immigration, health services, health status and women’s health (Fig. 1).

### Statistical analyses

Statistical analyses used the StataSE/13 software package. First, basic descriptive information, frequencies, and distributions, were explored by country of origin. We detected several interactions (*p* < 0.15); the interaction of country of origin with the length of stay in Finland (*p* = 0.0326), literacy (*p* = 0.0268), and marital status (*p* = 0.1054). We conducted further analyses separately for each country of origin. We produced age-standardized proportions (%) using predictive margins [32]. For the continuous variables, we calculated means and standard deviations (SDs) among those who responded to the question on screening participation in the previous five years (*n* = 620). Next, we analyzed age-adjusted bivariate associations between the screening participation in the previous five years and potential explanatory variables by country of origin. Logistic regression analyses provided Odds Ratios (ORs), 95% Confidence Intervals (CIs), and *p*-values.

Based on the age-adjusted results, we created four models for each immigrant group, considering factors from the previous stage, using alpha level *p* < 0.15 as the threshold. We then grouped these models; for instance, for the model representing socio-demographic factors, we selected the most significant variables for each immigrant group. We did the same for the models representing factors related to immigration, health services use, and health status. Thus, in the analyses, variables within one variable group (for example, socio-demographic) were adjusted for age plus each other. To create a final parsimonious and robust multiple-adjusted model, from the results of the previous analyses, we chose only those factors with a statistical significance level of *p* < 0.15. Thus, all the variables included in the final model were simultaneously adjusted for each other. To avoid multi-correlation problems, before creating any multiple-adjusted models, we checked correlations within potential explanatory variables. If there were high (*r* > 0.60) correlations, we included only one of those highly correlating variables simultaneously in the same model.

All our analyses involved Finite Population Correction (FPC) and the effects of non-response were statistically corrected with Inverse Probability Weighting (IPW) [33]. Potential predictors of non-response were the migrant group, age group, sex, city, and marital status. Using the Bayesian Information Criterion (BIC), the best-fitting model was chosen, and it included all the main effects of the covariates. Interactions between variables little improved the predicting power of the model. Afterwards the model to IPW was applied; commonly utilized in correcting the effects of non-response. To obtain representative aggregated results across cities, we further calibrated the IPW weights with respect to the population sizes of the strata.

## Results

### Descriptive results

Table 1 illustrates the main characteristics of the study participants. The mean age of Russians was the highest (42); their employment outside the home was relatively high (63%), and so was their Finnish or Swedish language proficiency (90%). However, a relatively low proportion were married or living in common-law partnership (63%). The mean age of migration was 30; about 82% of them lived in the metropolitan area Helsinki, Espoo, and Vantaa, and 63% had lived in Finland for ten years or longer. They reported similar rates of discrimination experiences (40%) as Kurds (39%) but higher rates compared with Somalis (17%).

**Table 1:** Characteristics of study participants by country of origin: weighted and age-adjusted proportions (%) or means and standard deviations (SD) among women who responded to the question of Pap test participation N/A = Not applicable (Question was not asked)

Characteristics		Total *n* of respondents	RUSSIAN (*n*=291) *n*(%)		SOMALI (*n*=132) *n*(%)		KURDISH (*n*=197) *n*(%)	
Socio-demographic category								
Age: Mean (SD)		620	42.3 (10.4)		39.3 (9.1)		38.4 (8.5)	
High school in any country		618	249	87.0	16	14.4	79	42.0
Literacy in Finnish / Swedish		596	254	90.7	76	71.3	152	79.8
Married or common law partnership		617	191	62.7	97	75.8	153	75.4
Main Religion	Christian for Russians	617	202	72.6	130	99.2	150	75.1
	Islam for Somali & Kurdish							
Number of family members: Mean (SD)		620	2.54 (1.1)		5.37 (2.8)		3.75 (1.6)	
Employment status	Housewife	616	30	13.5	48	42.7	33	18.2
	Employed	616	189	62.8	46	30.4	96	48.8
Living outside of the metropolitan area of Finland		620	187	81.0	94	89.7	116	55.0
Immigration category								
Age of migration: Mean (SD)		618	30.2 (10.7)		27.6 (9.8)		26.8 (8.8)	
Living in Finland for ten years or longer		618	188	63.4	81	68.7	122	61.6
Has had discrimination experience		620	291	40.0	132	17.0	197	39.0
Health care services category								
Pap test taken (previous five years)		620	219	73.9	40	34.7	121	61.3
At least one general health check-up (previous five years)		616	261	92.2	69	57.7	152	77.2
At least one gynecological check-up (previous five years)		616	213	73.8	34	30.5	97	50.1
Distrust in public health care services		614	123	41.1	26	23.7	53	27.4
Health category								
Very good or good self-rated health status		620	166	64.4	104	78.8	111	58.3
Has ever given birth		608	235	74.6	111	89.9	180	90.9
Has had female genital mutilation (FGM)		309	N/A		93	79.0	67	36.0
Has experienced problems due to FGM		155	N/A		16	21.8	22	33.6

Among Somalis, the mean age was 39; the mean number of family members were 5.4 (compared with 3.8 for Kurds and 2.5 for Russians). The mean age of migration was 28; about 90% of them lived in the metropolitan area, while some 70% had lived in Finland for ten years or longer. Almost all of them reported Islam as their main religion (99%), 75% Kurds also reported Islam, while 91% Russians were Christian. Somalis had relatively good self-rated health status (79%), compared with 58% in Kurds and 64% in Russians. Only 14% Somalis had high school education, compared with 87% Russians and 42% Kurds.

Kurdish women were the youngest (average 38), and the mean age of migration was 27. Of them, 55% lived in metropolitan area, while 62% had lived in Finland for ten years or longer. This group had almost the same proportion of those having given birth at least once (91%, compared with 90% Somalis). We had 34% Kurds and 22% Somalis reporting problems due to FGM. Questions on FGM were targeted at those with evidence of prevalence in their country of origin based on UNICEF Statistics [34], hence, Russians were excluded.

Table 1 displays the weighted age-adjusted proportions of screening participation by country of origin. Significant differences existed between these countries (*p* < 0.001). The participation rate was highest among Russians [73.9% (95% CI = 68.1-79.7)], followed by Kurds [61.3% (55.0-67.7)]; Somalis had the lowest [34.7% (26.4-43.0)].

### Factors associated with participation in cervical cancer screening

Table 2 illustrates the age-adjusted bivariate associations between potential factors and participation in screening in the previous five years by country of origin. When adjusted for age, the significant factor for higher screening participation common to all groups was having had at least one general health exam (p-values ranging from 0.025 to <0.001) or gynecological check-up (*p* < 0.001) in the previous five years.

**Table 2:** Age-adjusted bivariate associations between the Pap test participation in the previous five years and explanatory variables by country of origin

Variables		RUSSIAN (*n*=291) OR (95% CI)	*p*	SOMALI (*n*=132) OR (95% CI)	*p*	KURDISH (*n*=197) OR (95% CI)	*p*
Socio-demographic category							
Age (continuous)		1.02(0.99-1.05)	0.218	1.02(0.98-1.06)	0.249	1.01(0.98-1.04)	0.527
High school in any country		1.09(0.48-2.46)	0.837	1.97(0.73-5.32)	0.181	1.53(0.87-2.69)	0.138
Literacy in Finnish / Swedish		4.04(1.76-9.23)	0.001	2.48(0.86-7.14)	0.091	1.19(0.60-2.35)	0.617
Married / common law partnership		0.98(0.53-1.82)	0.951	1.70(0.65-4.40)	0.276	2.50(1.32-4.73)	0.005
Main religion	Christian for Russians	0.79(0.41-1.52)	0.488	N/E		1.36(0.70-2.64)	0.367
	Islam for Somali & Kurdish						
Number of family members (Continuous)		1.38(1.04-1.84)	0.026	1.09(0.96-1.25)	0.164	1.26(1.04-1.53)	0.018
Employment status	Housewife	1.86(0.62-5.60)	0.266	0.77(0.29-2.05)	0.604	2.23(0.93-5.38)	0.073
	Employed	1.89(0.96-3.70)	0.065	0.63(0.24-1.66)	0.350	3.31(1.79-6.11)	<0.001
Living outside the metropolitan area of Finland		1.33(0.73-2.41)	0.355	0.11(0.03-0.46)	0.002	1.96(1.11-3.46)	0.021
Immigration category							
Age of migration (continuous)		0.92(0.88-0.98)	0.005	0.95(0.90-1.02)	0.162	1.00(0.94-1.05)	0.916
Living in Finland for ten years or longer		2.14(1.14-4.03)	0.018	1.28(0.57-2.87)	0.551	0.95(0.54-1.69)	0.870
Has experienced any discrimination		2.19(1.16-4.14)	0.016	0.85(0.32-2.25)	0.744	1.17(0.67-2.04)	0.588
Health care services category							
At least one general health check-up (previous five years)		3.38(1.53-7.48)	0.003	2.58(1.13-5.92)	0.025	3.40(1.76-6.56)	<0.001
At least one gynecological check-up (previous five years)		8.38(4.19-16.8)	<0.001	11.8(4.69-29.5)	<0.001	23.5(11.1-49.7)	<0.001
Distrust in public healthcare services		1.22(0.66-2.25)	0.533	0.25(0.08-0.74)	0.520	0.83(0.45-1.53)	0.560
Health category							
Very good or good self-rated health status		1.20(0.64-2.23)	0.565	1.36(0.53-3.46)	0.520	1.58(0.88-2.82)	0.123
Has ever given birth		1.80(0.84-3.88)	0.131	1.46(0.39-5.47)	0.725	8.15(2.33-28.5)	0.001
Has had Female genital mutilation (FGM)		N/A		0.19(0.45-3.16)	0.725	1.08(0.60-1.93)	0.794
Has experienced problems due to FGM		N/A		0.21(0.05-0.93)	0.041	1.23(0.44-3.47)	0.694

*N/E* Not estimated, *N/A* Not applicable (Question was not asked)

Table 3 displays the results of the first multiple logistic regression models; the estimates within each of the four variable groups are adjusted for age plus each other. Here, the only significant common factor was having had a gynecological check-up in the previous five years (*p* < 0.001).

**Table 3:** Multiple logistic regression models adjusted simultaneously for variables within each main category ^a^

Variables	RUSSIAN OR (95% CI)	*p*	SOMALI OR (95% CI)	*p*	KURDISH OR (95% CI)	*p*
Socio-demographic category	(*n* = 286)^b^		(*n* = 17)^b^		(*n* = 196)^b^	
Age (continuous)	1.03(0.99-1.06)	0.092	1.05(0.99-1.10)	0.089	1.03(0.99-1.07)	0.107
High school in any country	N/A		N/A		1.86(1.01-3.44)	0.047
Literacy in Finnish / Swedish	3.80(1.60-9.01)	0.002	2.38(0.78-7.31)	0.129	N/A	
Married/common law partnership	N/A		N/A		1.98(0.93-4.21)	0.075
Number of family members (Continuous)	1.38(1.00-1.91)	0.047	N/A		1.23(1.01-1.59)	0.038
Employment status	Housewife	1.32(0.36-4.86)	0.680	N/A	1.67(0.69-4.05)	0.257
	Employed	1.69(0.84-3.37)	0.137	N/A	3.35(1.73-6.45)	<0.001
Living outside of the metropolitan area of Finland	N/A		0.10(0.02-0.44)	0.002	2.29(1.28-4.11)	0.005
Immigration category	(*n* = 291)^b^		(N/A)		(N/A)	
Age (continuous)	1.12(1.01-1.24)	0.029	N/A		N/A	
Age of migration (continuous)	0.91(0.82-1.00)	0.042	N/A		N/A	
Living in Finland for ten years or longer	0.65(0.21-1.94)	0.437	N/A		N/A	
Has experienced any discrimination	2.01(1.03-3.93)	0.040	N/A		N/A	
Health care services category	(*n* = 289)^b^		(*n* = 129)^b^		(*n* = 194)^b^	
Age (continuous)	1.04(1.00-1.07)	0.042	1.03(0.98-1.09)	0.186	1.02(0.99-1.06)	0.191
At least one general health check-up (previous five years)	1.81(0.75-4.36)	0.182	1.40(0.53-3.65)	0.495	2.15(1.05-4.40)	0.035
At least one gynecological check-up (previous five years)	7.55(3.73-15.3)	<0.001	9.38(3.56-24.7)	<0.001	20.9(9.84-44.5)	<0.001
Distrust in public healthcare services	N/A		0.31(0.09-1.12)	0.073	N/A	
Health category	(*n* = 291)^b^		(*n* = 113)^b^		(*n* = 195)^b^	
Age (continuous)	1.01(0.97-1.04)	0.600	1.03(0.98-1.07)	0.207	1.01(0.97-1.05)	0.586
Very good or good self-rated health status	N/A		N/A		1.64(0.90-2.99)	0.108
Has ever given birth	1.80(0.84-3.88)	0.131	N/A		8.05(2.18-29.7)	0.002
Has experienced problems due to FGM	N/A		0.23(0.53-1.01)	0.051	N/A	

^a^ = In each category model, all variables included are simultaneously adjusted for each other

^b^ = Number of observations with non-missing value in all variables in the model

*N/A* Not applicable, because this variable was not included in the model

Table 4 illustrates the results of the final multiple logistic regression models; the estimates are adjusted simultaneously for all other variables in each model. Among Russians, in addition to age, significant factors (*p* < 0.05) associated with higher likelihood of screening participation were literacy in Finnish/Swedish (*p* = 0.003) and gynecological health check-ups in the previous five years (*p* < 0.001). Discrimination experiences (*p* = 0.085) and higher age of immigration (*p* = 0.104) approached statistical significance.

**Table 4:** Final multiple logistic regression models for each country of origin

Variables	RUSSIAN (*n*=284) OR (95% CI)	*p*	SOMALI (*n*=98) OR (95% CI))	*p*	KURDISH (*n*=191) OR (95% CI)	*p*
Age (Continuous)	1.10(1.02-1.18)	0.015	1.06(0.99–1.13)	0.074	1.02(0.98-1.07)	0.325
High school in any country	N/A		N/A		2.63(1.22-5.67)	0.014
Literacy in Finnish/ Swedish	3.63(1.53-8.60)	0.003	2.58(0.83-8.07)	0.102	N/A	
Married / common law partnership	N/A		N/A		0.69(0.25-1.89)	0.468
Number of family members (continuous)	1.33(0.82-2.14)	0.244	N/A		1.23(0.95-1.58)	0.113
Employed	1.64(0.73-3.73)	0.230	N/A		4.31(1.49-12.5)	0.007
Living outside of the metropolitan area of Finland	N/A		0.15(0.02-0.87)	0.035	0.77(0.34-1.71)	0.517
Age of migration (continuous)	0.95(0.89-1.01)	0.104	N/A		N/A	
Has experienced any discrimination	1.91(0.91-3.99)	0.085	N/A		N/A	
At least one general health check-up (previous five years)	N/A		N/A		1.86(0.87-3.98)	0.109
At least one gynecological check-up (previous five years)	9.69(4.52-20.7)	<0.001	6.54(2.15-19.8)	<0.001	26.2(11.4-60.1)	<0.001
Distrust in public health care services	N/A		0.33(0.09-1.26)	0.105	N/A	
Very good or good self-rated health status	N/A		N/A		0.81(0.31-2.14)	0.676
Has ever given birth	0.96(0.28-3.29)	0.953	N/A		9.34(1.58-55.1)	0.014
Has experienced problems due to FGM	N/A		0.67(0.15-3.01) 0.601		N/A	

*N/A* Not applicable, because variable was not included in the model

Among Somalis, the significant factor associated with higher likelihood of screening participation was having had at least one gynecological check-up in the previous five years (*p* < 0.001); living outside of metropolitan area significantly decreased the likelihood of participation (*p* = 0.035).

Among Kurds, factors increasing the likelihood of screening participation were as follows: high school education (*p* = 0.014), being employed (*p* = 0.007), having given birth at least once (*p* = 0.014), and having had a gynecological check-up in the previous five years (*p* < 0.001).

## Discussion

The aim of this study was to explore factors associated with participation in cervical cancer screening among immigrant women of Russian, Somali, and Kurdish origin living in Finland. We included women aged 25-60 for the analyses because, in Finland, all women are usually invited to cervical cancer screening from age 25 or mostly from 30 until 60 or 65, depending on the municipality [3]. Women participate in mass screening, opportunistic testing, or both. Consistent with previous results [35], the most significant factor for screening participation among all the groups was having had gynecological check-ups in the previous five years. Individuals’ general physicians or gynecologists have a vital role to play in referring women to preventive care services or advising their utilization [13, 36, 37]. Another factor for higher screening participation common among women with Russian and Kurdish origins was having given birth at least once. However, in the final model, this factor was significant only for Kurdish women. Women who do not use reproductive health services might need more information on the importance of screening [21, 22].

In general, Russians were the most active in cervical cancer screening participation. One facilitating factor was literacy in Finnish/Swedish, the official languages of Finland. Studies have highlighted poor language proficiency as one of the most significant barriers to screening participation among immigrants [15, 17, 38]. Adequate communication skills promote accessibility to healthcare services such as screening. Hence, it is crucial that recipients can read and understand the content of the screening invitation letter in the official language of the country and communicate with healthcare professionals [39]. Another factor was a long period of stay in Finland (minimum ten years), consistent with previous studies [9, 15] yet, it turned non-significant when adjusted for other immigration-related variables. Staying long enough in the host country enables immigrants to get acquainted with the healthcare system. Having many members in the family was associated with higher participation, consistent with an earlier study [23]. However, this factor became non-significant when adjusted for other socio-demographic factors. For Russians, one barrier was a high age of migration; it remained almost significant, even in the final model; this finding is consistent with previous studies [9, 11]. Young immigrants have a better chance of acquiring language skills mainly through schools, where they are more likely to be exposed to health promotion, including information about the purposes of screening.

Somalis reported the lowest participation rate, consistent with an earlier finding that Somali and Muslim women participate less in cervical cancer screening and other screenings compared with other immigrant groups or non-immigrants [40]. Language problems may exist, leading to increased need to use medical interpreters among this group [17, 40]. Living outside of metropolitan area was a barrier among Somalis in our study. These women might have difficulties in accessing screening sites due to longer distances and less public transportation; these results confirm similar studies [31, 41]. Another potential barrier among Somalis was having had FGM-related problems. However, when adjusted for other factors, this factor became non-significant in the final model. This aspect may be ascribed to lack of statistical power, with only a small number of Somali women reporting problems. Specifically, women with FGM may experience discomfort and embarrassment during a gynecological examination [17], which can constitute as a barrier to screening. Another barrier in our initial analysis, which became non-significant in the final model, was distrust in public healthcare. This can be associated with lack of trust in physicians or other healthcare staff, as indicated in a previous study [16].

Among Kurdish women, higher education level and being married or in a common-law partnership were associated with greater participation in cervical cancer screening, in line with previous studies [15, 19, 35, 42]. This aspect might reflect the role of husbands/partners as well as utilization of reproductive healthcare services [43]. Although, in Finland, the organized screening program is free of charge to all eligible women, being employed was associated with screening participation among Kurds, as demonstrated in earlier studies [20, 44]. Some members of this group may be somewhat unaware of the screening program, the healthcare system in Finland, and the purpose of preventive healthcare services, which are explained in occupational healthcare to employees.

### Strengths and limitations of the study

The strengths of this study are that it has a population-based design and satisfactory response rates among Russian and Kurdish participants. To promote willingness to respond to gender-specific issues, the interviewers were mostly female. Bilingual interviewers spoke the same language as the participants, thus promoting understanding about the study purpose and specific interview questions [29]. The study has some limitations. Screening participation in the previous five years was self-reported; as such, some recall bias [45, 46] or reporting errors with participants unfamiliar with screening might exist. Further, some interviews had male interviewers when a female interviewer was unavailable; this may have caused some reporting bias or item-specific non-response, especially in questions related to reproductive health. However, the interviewers were trained to specify unfamiliar terms where necessary.

Among Somalis, the response rate to the entire survey and item response to screening participation were lower in comparison to Russian and Kurdish women. This feature limited the statistical power of the analysis and might have produced some bias in the study results. It may also explain some of the non-significant associations in this study. We observed wide Confidence Intervals, especially among Kurds on the variable ‘Has ever given birth.’ Among Kurdish women, 90.9% had given birth, which means that only a few (*n* = 15) had never given birth. Among these 15 women, only three reported having had a Pap test taken. Thus, this variable may be underpowered in our multiple adjusted analysis.

The variation in screening participation among the immigrant groups might be partially due to factors such as cultural beliefs and norms, which were not included in the data collection for this study. A qualitative research with an ethnographic approach [47, 48] might be successful in studying such factors, especially among Somali women. An ethnographic method would provide a deeper understanding of Somali women’s participation barriers and thus an opportunity to support their screening participation.

These limitations notwithstanding, this study has succeeded, for the first time in Finland, in identifying some enabling factors and barriers associated with screening participation among immigrant groups. This study demonstrates that, although Finland, like some other European countries [11, 20, 25, 26] offers ‘cost-free’ screening targeting all eligible women, disparities exist in using this service among different immigrant groups. These results are worth considering when planning interventions to enhance screening participation among immigrants.

## Conclusion

Participating in cervical cancer screening was facilitated and hindered mostly by different factors among the immigrant groups in this study. Our results suggest that women who refrain from using reproductive health services and those unemployed and with low education might need more information on the importance of screening participation. Primary and occupational healthcare services may have a significant role in informing immigrant women about this importance. In addition to the factors identified in our study, others such as cultural beliefs and norms might explain why some immigrants seldom participate in cervical cancer screening. To learn more about these cultural factors and health-related beliefs, qualitative studies with different approaches are imperative.
